# Supplementary material for: Using authentic representations of practice in teacher education: Do direct instructional and problem-based approaches really produce different effects?
Source: PLoS One. 2022 Sep 2;17(9):e0273988. doi: 10.1371/journal.pone.0273988 (PMC9439238; doi:10.1371/journal.pone.0273988)
Supplement: S1 File — (HTML) [file pone.0273988.s001.html]

Supplement


Code 

- Show All Code
- Hide All Code

# Supplement

### On the journal article …

#### Jürgen Schneider

University of Tübingen  
juergen.schneider@uni-tuebingen.de

#### 2022-07-19

# system properties

**Used packages**

```
knitr::opts_chunk$set(echo = T, message = F, warning = F)
library(rio)
library(tidyverse)
library(stringr)
library(mice)
library(miceadds)
library(naniar)
library(corrgram)
library(lavaan)
library(pander)
library(bain)
library(MASS)
library(BayesFactor)
library(ggthemes)
library(psych)
library(ggside)
```

**R-Version**

```
R.Version()
```

```
## $platform
## [1] "x86_64-w64-mingw32"
## 
## $arch
## [1] "x86_64"
## 
## $os
## [1] "mingw32"
## 
## $system
## [1] "x86_64, mingw32"
## 
## $status
## [1] ""
## 
## $major
## [1] "4"
## 
## $minor
## [1] "0.2"
## 
## $year
## [1] "2020"
## 
## $month
## [1] "06"
## 
## $day
## [1] "22"
## 
## $`svn rev`
## [1] "78730"
## 
## $language
## [1] "R"
## 
## $version.string
## [1] "R version 4.0.2 (2020-06-22)"
## 
## $nickname
## [1] "Taking Off Again"
```

# Import data

- **Data “ZA6259\_v1-0-0.sav”**
  - Get the **open data** via the repository https://doi.org/10.4232/1.13468
  - or by clicking here to download the Data that are embedded in this HTML file
- **Data “ts.Rdata”**: Click here to download the Data that are embedded in this HTML file
- **Analyses**: Github Repo https://github.com/j-5chneider/pbl-vs-di

```
# this is the data you need to download from Gesis or from within this file
pbl_di <- rio::import("data_public/ZA6259_v1-0-0.sav")
```

# Wrangle data

```
# construct treatment variable
# 0 = DI, 1 = PBL
pbl_di$treatment <- ifelse(c(pbl_di$seminartyp == 4 | pbl_di$seminartyp == 2), 0, 1)


# get variables on theory and selection from other data set (needs to be reintegrated into original data set)
ts <- rio::import("data_public/ts.Rdata") %>%
  dplyr::select(code, anz.komm.43.1, anz.komm.43.2, anz.komm.43.7, anz.komm.43.3, anz.komm.43.4, anz.komm.43.5, 
                theorie.r.43.1, theorie.r.43.2, theorie.r.43.7, theorie.r.43.3, theorie.r.43.4, theorie.r.43.5,
                doz_pass, doz_gef) %>%
  dplyr::mutate(code = str_trim(code))

pbl_di <- left_join(pbl_di, ts, by ="code")


# construct centered variable for attendance
pbl_di <- pbl_di %>%
  dplyr::mutate(attendance = rowSums(data.frame(anwesend_erst, anwesend_zweit)))

# anwesend_MEAN <- pbl_di %>%
#                     summarize(anwesend_M = mean(anwesend, na.rm=TRUE))

# construct variable literature
pbl_di <- pbl_di %>%
  dplyr::mutate(literature = rowSums(data.frame(T2_text_1, T2_text_2, T2_text_3)))

# construct the variable lit_pre (literature read before the treatment)
pbl_di <- pbl_di %>%
  dplyr::mutate(lit_pre = rowSums(data.frame(T1_text_1, T1_text_2, T1_text_3)))

# construct the variable prior_knowledge, 
# based on a test on declarative knowledge on classroom management
pbl_di <- pbl_di %>%
  mutate(prior_knowledge = rowMeans(data.frame(T1_wiss_crm_verh1, T1_wiss_crm_verh2, T1_wiss_crm_verh3, T1_wiss_crm_verh4, T1_wiss_crm_verh5, T1_wiss_crm_verh6, T1_wiss_crm_bez1, T1_wiss_crm_bez2, T1_wiss_crm_unt1, T1_wiss_crm_unt2, T1_wiss_crm_unt3, T1_wiss_crm_unt4, T1_wiss_crm_unt5, T1_wiss_crm_unt6), na.rm = T))


pbl_di <- pbl_di %>%
  mutate(sel_att_pre1 = case_when(
                           is.na(theorie.r.43.1) & is.na(theorie.r.43.2) & is.na(theorie.r.43.7) &
                           is.na(A43_1) & is.na(A43_2) & is.na(A43_7) ~ as.numeric(NA),
                           TRUE ~ as.numeric(anz.komm.43.1)),
         sel_att_pre2 = case_when(
                           is.na(theorie.r.43.1) & is.na(theorie.r.43.2) & is.na(theorie.r.43.7) &
                           is.na(A43_1) & is.na(A43_2) & is.na(A43_7) ~ as.numeric(NA),
                           TRUE ~ as.numeric(anz.komm.43.2)),
         sel_att_pre3 = case_when(
                           is.na(theorie.r.43.1) & is.na(theorie.r.43.2) & is.na(theorie.r.43.7) &
                           is.na(A43_1) & is.na(A43_2) & is.na(A43_7) ~ as.numeric(NA),
                           TRUE ~ as.numeric(anz.komm.43.7)),
         sel_att_post1 = case_when(
                           is.na(theorie.r.43.3) & is.na(theorie.r.43.4) & is.na(theorie.r.43.5) &
                           is.na(A43_3) & is.na(A43_4) & is.na(A43_5) ~ as.numeric(NA),
                           TRUE ~ as.numeric(anz.komm.43.3)),
         sel_att_post2 = case_when(
                           is.na(theorie.r.43.3) & is.na(theorie.r.43.4) & is.na(theorie.r.43.5) &
                           is.na(A43_3) & is.na(A43_4) & is.na(A43_5) ~ as.numeric(NA),
                           TRUE ~ as.numeric(anz.komm.43.4)),
         sel_att_post3 = case_when(
                           is.na(theorie.r.43.3) & is.na(theorie.r.43.4) & is.na(theorie.r.43.5) &
                           is.na(A43_3) & is.na(A43_4) & is.na(A43_5) ~ as.numeric(NA),
                           TRUE ~ as.numeric(anz.komm.43.5))
         )


# deleting some variables to avoid problems with imputation
pbl_di <- pbl_di %>%
  mutate(theory_pre1 = theorie.r.43.1,
         theory_pre2 = theorie.r.43.2,
         theory_pre3 = theorie.r.43.7,
         theory_post1 = theorie.r.43.3,
         theory_post2 = theorie.r.43.4,
         theory_post3 = theorie.r.43.5,
         inquiry_pre1 = A43_1, 
         inquiry_pre2 = A43_2, 
         inquiry_pre3 = A43_7, 
         inquiry_post1 = A43_3, 
         inquiry_post2 = A43_4, 
         inquiry_post3 = A43_5) %>% # rename some variables for easier understanding
  dplyr::select(treatment, seminar, attendance, 
         sel_att_pre1, sel_att_pre2, sel_att_pre3, sel_att_post1, sel_att_post2, sel_att_post3,
         theory_pre1, theory_pre2, theory_pre3, theory_post1, theory_post2, theory_post3, 
         inquiry_pre1, inquiry_pre2, inquiry_pre3, inquiry_post1, inquiry_post2, inquiry_post3, literature,
         T2_anstrS_1, T2_anstrS_2, T2_anstrS_3, T2_anstrT_1, T2_anstrT_2, T2_anstrT_3, T2_anstrT_4,
         doz_pass, doz_gef,
         geschl, unterrichtet, erf_vid, lit_pre, prior_knowledge) %>%
  dplyr::filter(!is.na(seminar))
```

# Names and labels of variables

```
names_labels <- data.frame(names = names(pbl_di[1:31]),
                          `item labels` = c(
                            "Treatment variable [0: DI, 1: PBL]",
                            "ID of course group (used as cluster variable)",
                            "How many of the treatment sessions the student attended [0,1,2]",
                            "Measure of selective attention, first sequence of pre-test [quantity of comments]",
                            "Measure of selective attention, second sequence of pre-test [quantity of comments]",
                            "Measure of selective attention, third sequence of pre-test [quantity of comments]",
                            "Measure of selective attention, first sequence of post-test [quantity of comments]",
                            "Measure of selective attention, second sequence of post-test [quantity of comments]",
                            "Measure of selective attention, third sequence of post-test [quantity of comments]",
                            "Measure of theory integration, first sequence of pre-test [% of comments with theory integration: 0-100]",
                            "Measure of theory integration, second sequence of pre-test [% of comments with theory integration: 0-100]",
                            "Measure of theory integration, third sequence of pre-test [% of comments with theory integration: 0-100]",
                            "Measure of theory integration, first sequence of post-test [% of comments with theory integration: 0-100]",
                            "Measure of theory integration, second sequence of post-test [% of comments with theory integration: 0-100]",
                            "Measure of theory integration, third sequence of post-test [% of comments with theory integration: 0-100]",
                            "Measure of inquiry steps realized, first sequence of pre-test [0,1,2,3]",
                            "Measure of inquiry steps realized, second sequence of pre-test [0,1,2,3]",
                            "Measure of inquiry steps realized, third sequence of pre-test [0,1,2,3]",
                            "Measure of inquiry steps realized, first sequence of post-test [0,1,2,3]",
                            "Measure of inquiry steps realized, second sequence of post-test [0,1,2,3]",
                            "Measure of inquiry steps realized, third sequence of post-test [0,1,2,3]",
                            "number of theoretical literature on classroom management read [0,1,2,3]",
                            "Willingness for effort, Likert item 1 [1,2,3,4]",
                            "Willingness for effort, Likert item 2 [1,2,3,4]",
                            "Willingness for effort, Likert item 3 [1,2,3,4]",
                            "Willingness for effort, Likert item 4 [1,2,3,4]",
                            "Willingness for effort, Likert item 5 [1,2,3,4]",
                            "Willingness for effort, Likert item 6 [1,2,3,4]",
                            "Willingness for effort, Likert item 7 [1,2,3,4]",
                            "Instructors beliefs about the treatment, item 1 [1,2,3,4,5,6]",
                            "Instructors beliefs about the treatment, item 2 [1,2,3,4,5,6]"
                          ))

panderOptions('table.alignment.default', 'left')
pander(names_labels)
```

| names | item.labels |
| --- | --- |
| treatment | Treatment variable [0: DI, 1: PBL] |
| seminar | ID of course group (used as cluster variable) |
| attendance | How many of the treatment sessions the student attended [0,1,2] |
| sel\_att\_pre1 | Measure of selective attention, first sequence of pre-test [quantity of comments] |
| sel\_att\_pre2 | Measure of selective attention, second sequence of pre-test [quantity of comments] |
| sel\_att\_pre3 | Measure of selective attention, third sequence of pre-test [quantity of comments] |
| sel\_att\_post1 | Measure of selective attention, first sequence of post-test [quantity of comments] |
| sel\_att\_post2 | Measure of selective attention, second sequence of post-test [quantity of comments] |
| sel\_att\_post3 | Measure of selective attention, third sequence of post-test [quantity of comments] |
| theory\_pre1 | Measure of theory integration, first sequence of pre-test [% of comments with theory integration: 0-100] |
| theory\_pre2 | Measure of theory integration, second sequence of pre-test [% of comments with theory integration: 0-100] |
| theory\_pre3 | Measure of theory integration, third sequence of pre-test [% of comments with theory integration: 0-100] |
| theory\_post1 | Measure of theory integration, first sequence of post-test [% of comments with theory integration: 0-100] |
| theory\_post2 | Measure of theory integration, second sequence of post-test [% of comments with theory integration: 0-100] |
| theory\_post3 | Measure of theory integration, third sequence of post-test [% of comments with theory integration: 0-100] |
| inquiry\_pre1 | Measure of inquiry steps realized, first sequence of pre-test [0,1,2,3] |
| inquiry\_pre2 | Measure of inquiry steps realized, second sequence of pre-test [0,1,2,3] |
| inquiry\_pre3 | Measure of inquiry steps realized, third sequence of pre-test [0,1,2,3] |
| inquiry\_post1 | Measure of inquiry steps realized, first sequence of post-test [0,1,2,3] |
| inquiry\_post2 | Measure of inquiry steps realized, second sequence of post-test [0,1,2,3] |
| inquiry\_post3 | Measure of inquiry steps realized, third sequence of post-test [0,1,2,3] |
| literature | number of theoretical literature on classroom management read [0,1,2,3] |
| T2\_anstrS\_1 | Willingness for effort, Likert item 1 [1,2,3,4] |
| T2\_anstrS\_2 | Willingness for effort, Likert item 2 [1,2,3,4] |
| T2\_anstrS\_3 | Willingness for effort, Likert item 3 [1,2,3,4] |
| T2\_anstrT\_1 | Willingness for effort, Likert item 4 [1,2,3,4] |
| T2\_anstrT\_2 | Willingness for effort, Likert item 5 [1,2,3,4] |
| T2\_anstrT\_3 | Willingness for effort, Likert item 6 [1,2,3,4] |
| T2\_anstrT\_4 | Willingness for effort, Likert item 7 [1,2,3,4] |
| doz\_pass | Instructors beliefs about the treatment, item 1 [1,2,3,4,5,6] |
| doz\_gef | Instructors beliefs about the treatment, item 2 [1,2,3,4,5,6] |

# Pre-treatment comparison

between the two treatment groups concerning…

## gender

```
d_preT <- pbl_di %>%
  dplyr::filter(!is.na(geschl) & !is.na(treatment))

ttestBF(formula = geschl ~ treatment,
        data = d_preT)
```

```
## Bayes factor analysis
## --------------
## [1] Alt., r=0.707 : 0.1087726 ±0.17%
## 
## Against denominator:
##   Null, mu1-mu2 = 0 
## ---
## Bayes factor type: BFindepSample, JZS
```

## teaching experience

```
d_preT <- pbl_di %>%
  dplyr::filter(!is.na(unterrichtet) & !is.na(treatment))

ttestBF(formula = unterrichtet ~ treatment,
        data = d_preT)
```

```
## Bayes factor analysis
## --------------
## [1] Alt., r=0.707 : 0.1155389 ±0.16%
## 
## Against denominator:
##   Null, mu1-mu2 = 0 
## ---
## Bayes factor type: BFindepSample, JZS
```

## experience with video based analysis

```
d_preT <- pbl_di %>%
  dplyr::filter(!is.na(erf_vid) & !is.na(treatment))

ttestBF(formula = erf_vid ~ treatment,
        data = d_preT)
```

```
## Bayes factor analysis
## --------------
## [1] Alt., r=0.707 : 0.09939937 ±0.18%
## 
## Against denominator:
##   Null, mu1-mu2 = 0 
## ---
## Bayes factor type: BFindepSample, JZS
```

## literature read

```
d_preT <- pbl_di %>%
  dplyr::filter(!is.na(lit_pre) & !is.na(treatment))

ttestBF(formula = lit_pre ~ treatment,
        data = d_preT)
```

```
## Bayes factor analysis
## --------------
## [1] Alt., r=0.707 : 0.1144645 ±0.16%
## 
## Against denominator:
##   Null, mu1-mu2 = 0 
## ---
## Bayes factor type: BFindepSample, JZS
```

## prior knowledge

```
d_preT <- pbl_di %>%
  dplyr::filter(!is.na(prior_knowledge) & !is.na(treatment))

ttestBF(formula = prior_knowledge ~ treatment,
        data = d_preT)
```

```
## Bayes factor analysis
## --------------
## [1] Alt., r=0.707 : 0.1011116 ±0.18%
## 
## Against denominator:
##   Null, mu1-mu2 = 0 
## ---
## Bayes factor type: BFindepSample, JZS
```

# Treatment check

Comparing several features between the treatment groups in both sessions respectively. Features are evaluated by trained raters attending and observing the instruction, disagreements were resolved through discussion.

## direct instruction from instructor

Should be different.

**First session**

```
treat_check <- rio::import(file = "data_public/rating-treatmentcheck.sav") %>%
  mutate(di_s1 = rowMeans(data.frame(B1_leitung_Doz1_u, B1_leitung_Doz2, B1_leitung_Doz3, B1_leitung_Doz4_u, B1_leitung_Doz5, B1_leitung_Doz6, B1_leitung_Doz7_u, B1_leitung_Doz8), na.rm = T),
         di_s2 = rowMeans(data.frame(B2_leitung_Doz1_u, B2_leitung_Doz2, B2_leitung_Doz3, B2_leitung_Doz4_u, B2_leitung_Doz5, B2_leitung_Doz6, B2_leitung_Doz7_u, B2_leitung_Doz8), na.rm = T),
         min_cases_s1 = B1_min_fallarb,
         min_cases_s2 = B2_min_fallarb,
         groups_s1 = B1_kleingruppen,
         groups_s2 = B2_kleingruppen,
         treatment = llm)

ttestBF(formula = di_s1 ~ treatment,
        data = treat_check)
```

```
## Bayes factor analysis
## --------------
## [1] Alt., r=0.707 : 3760694 ±0%
## 
## Against denominator:
##   Null, mu1-mu2 = 0 
## ---
## Bayes factor type: BFindepSample, JZS
```

**Second session**

```
ttestBF(formula = di_s2 ~ treatment,
        data = treat_check)
```

```
## Bayes factor analysis
## --------------
## [1] Alt., r=0.707 : 233620.7 ±0%
## 
## Against denominator:
##   Null, mu1-mu2 = 0 
## ---
## Bayes factor type: BFindepSample, JZS
```

## minutes spent on case-based learning

Should be equal.

**First session**

```
ttestBF(formula = min_cases_s1 ~ treatment,
        data = treat_check)
```

```
## Bayes factor analysis
## --------------
## [1] Alt., r=0.707 : 0.5137568 ±0%
## 
## Against denominator:
##   Null, mu1-mu2 = 0 
## ---
## Bayes factor type: BFindepSample, JZS
```

**Second session**

```
ttestBF(formula = min_cases_s2 ~ treatment,
        data = treat_check)
```

```
## Bayes factor analysis
## --------------
## [1] Alt., r=0.707 : 0.5452981 ±0%
## 
## Against denominator:
##   Null, mu1-mu2 = 0 
## ---
## Bayes factor type: BFindepSample, JZS
```

## worked in small groups

Should be different.

**First session**

```
ttestBF(formula = groups_s1 ~ treatment,
        data = treat_check)
```

```
## Bayes factor analysis
## --------------
## [1] Alt., r=0.707 : 567118.2 ±0%
## 
## Against denominator:
##   Null, mu1-mu2 = 0 
## ---
## Bayes factor type: BFindepSample, JZS
```

**Second session**

```
ttestBF(formula = groups_s2 ~ treatment,
        data = treat_check)
```

Vectors are constant (in the expected direction) that’s why we can’t compute a BF.

# Handle Missings

## Check for missingness

### Missing in data set

```
# save variables for descriptive statistics later on
pbl_di_desc <- pbl_di %>%
  dplyr::select(treatment, attendance, 
         sel_att_pre1, sel_att_pre2, sel_att_pre3, sel_att_post1, sel_att_post2, sel_att_post3,
         theory_pre1, theory_pre2, theory_pre3, theory_post1, theory_post2, theory_post3, 
         inquiry_pre1, inquiry_pre2, inquiry_pre3, inquiry_post1, inquiry_post2, inquiry_post3, literature,
         doz_pass, doz_gef,
         geschl, unterrichtet, erf_vid, lit_pre, prior_knowledge)

# delete not needed for imputation
pbl_di <- pbl_di %>%
  dplyr::select(-c(unterrichtet, erf_vid, lit_pre, prior_knowledge))

vis_miss(pbl_di)
```

### Patterns of missingness

```
gg_miss_upset(pbl_di)
```

## Decisions for imputation

### Number of imputations

1000

```
M <- 1000
```

### Imuptation model

for every variable

```
pbl_di$sel_att_pre1 <- as.numeric(pbl_di$sel_att_pre1)
pbl_di$sel_att_pre2 <- as.numeric(pbl_di$sel_att_pre2)
pbl_di$sel_att_pre3 <- as.numeric(pbl_di$sel_att_pre3)
pbl_di$sel_att_post1 <- as.numeric(pbl_di$sel_att_post1)
pbl_di$sel_att_post2 <- as.numeric(pbl_di$sel_att_post2)
pbl_di$sel_att_post3 <- as.numeric(pbl_di$sel_att_post3)
pbl_di$T2_anstrT_4 <- as.numeric(pbl_di$T2_anstrT_4)
pbl_di$geschl[pbl_di$geschl == 3] <- NA
pbl_di <- pbl_di %>%
  mutate(geschl = ifelse(geschl == 2, 1, 0))


# Define table
model_tab <- data.frame(variable = c("treatment", "seminar", 
                                     "attendance", "sel_att_pre1", "sel_att_pre2", 
                                     "sel_att_pre3", "sel_att_post1","sel_att_post2", 
                                     "sel_att_post3", "theory_pre1", "theory_pre2", 
                                     "theory_pre3", "theory_post1", "theory_post2", 
                                     "theory_post3", "inquiry_pre1", "inquiry_pre2", 
                                     "inquiry_pre3", "inquiry_post1", "inquiry_post2", 
                                     "inquiry_post3", "literature", "T2_anstrS_1", 
                                     "T2_anstrS_2", "T2_anstrS_3", "T2_anstrT_1", 
                                     "T2_anstrT_2","T2_anstrT_3", "T2_anstrT_4", 
                                     "doz_pass", "doz_gef", "geschl"),
                        'scale type' = c("binary", "nominal",
                                         "metric", "metric", "metric",
                                         "metric", "metric", "metric",
                                         "metric", "metric", "metric",
                                         "metric", "metric", "metric",
                                         "metric", "metric", "metric",
                                         "metric", "metric", "metric",
                                         "metric", "metric", "metric",
                                         "metric", "metric", "metric",
                                         "metric", "metric", "metric",
                                         "metric", "metric", "binary"),
                        method = c("logreg", "polyreg",
                                   "pmm", "pmm", "pmm",
                                   "pmm", "pmm", "pmm",
                                   "pmm", "pmm", "pmm",
                                   "pmm", "pmm", "pmm",
                                   "pmm", "pmm", "pmm",
                                   "pmm", "pmm", "pmm",
                                   "pmm", "pmm", "pmm",
                                   "pmm", "pmm", "pmm",
                                   "pmm", "pmm", "pmm",
                                   "pmm", "pmm", "polyreg"))

ini <- mice(pbl_di, 
            maxit = 0, 
            m = M,
            seed = 666
            )

# Define methods

meth <- ini$method

meth[1:32] <- c("logreg", "polyreg",
                "pmm", "pmm", "pmm",
                "pmm", "pmm", "pmm",
                "pmm", "pmm", "pmm",
                "pmm", "pmm", "pmm",
                "pmm", "pmm", "pmm",
                "pmm", "pmm", "pmm",
                "pmm", "pmm", "pmm",
                "pmm", "pmm", "pmm",
                "pmm", "pmm", "pmm",
                "pmm", "pmm", "logreg")

pander(model_tab)
```

| variable | scale.type | method |
| --- | --- | --- |
| treatment | binary | logreg |
| seminar | nominal | polyreg |
| attendance | metric | pmm |
| sel\_att\_pre1 | metric | pmm |
| sel\_att\_pre2 | metric | pmm |
| sel\_att\_pre3 | metric | pmm |
| sel\_att\_post1 | metric | pmm |
| sel\_att\_post2 | metric | pmm |
| sel\_att\_post3 | metric | pmm |
| theory\_pre1 | metric | pmm |
| theory\_pre2 | metric | pmm |
| theory\_pre3 | metric | pmm |
| theory\_post1 | metric | pmm |
| theory\_post2 | metric | pmm |
| theory\_post3 | metric | pmm |
| inquiry\_pre1 | metric | pmm |
| inquiry\_pre2 | metric | pmm |
| inquiry\_pre3 | metric | pmm |
| inquiry\_post1 | metric | pmm |
| inquiry\_post2 | metric | pmm |
| inquiry\_post3 | metric | pmm |
| literature | metric | pmm |
| T2\_anstrS\_1 | metric | pmm |
| T2\_anstrS\_2 | metric | pmm |
| T2\_anstrS\_3 | metric | pmm |
| T2\_anstrT\_1 | metric | pmm |
| T2\_anstrT\_2 | metric | pmm |
| T2\_anstrT\_3 | metric | pmm |
| T2\_anstrT\_4 | metric | pmm |
| doz\_pass | metric | pmm |
| doz\_gef | metric | pmm |
| geschl | binary | polyreg |

### Selection of predictors

We included the variables on `willingness for effort` for better prediction of missingness.

Check for multicollinearity:

```
corrgram(pbl_di, lower.panel = "panel.pie", upper.panel = "panel.cor")
```

The following variables are excluded as predictors: none.

```
# set predictor specifications
pred <- ini$predictorMatrix
# pred[,"stunde"] <- 0 # stunde highly correlated with beg_hw_min
```

**variables that are function of other variables**

**which variables to impute**  
All.

**number of iterations**  
20

```
maxit <- 20
```

## imputation

```
imp <- mice(pbl_di, 
            maxit = maxit, 
            m = M,
            meth = meth,
            pred = pred,
            seed = 666,
            printFlag = F
            )
```

## check imputation

**plausible values**  
of first 10 imputations

```
imp_conv <- mice(pbl_di, 
            maxit = maxit, 
            m = 10,
            meth = meth,
            pred = pred,
            seed = 666,
            printFlag = F
            )
lattice::stripplot(imp_conv, pch = 20, cex = 1.2)
```

All values seem plausible.

**check convergence**

of first 10 imputations

```
plot(imp_conv)
```

# Results

## Descriptive Statistics

Based on observed (and not imputed) data.  
**Group 0: DI**

```
# compute scales
pbl_di_desc <- pbl_di_desc %>%
  mutate(sel_att_pre = rowMeans(data.frame(sel_att_pre1, sel_att_pre2, sel_att_pre3), na.rm=T),
         sel_att_post = rowMeans(data.frame(sel_att_post1, sel_att_post2, sel_att_post3), na.rm=T),
         theory_pre = rowMeans(data.frame(theory_pre1, theory_pre2, theory_pre3), na.rm=T),
         theory_post = rowMeans(data.frame(theory_post1, theory_post2, theory_post3), na.rm=T),
         inquiry_pre = rowMeans(data.frame(inquiry_pre1, inquiry_pre2, inquiry_pre3), na.rm=T),
         inquiry_post = rowMeans(data.frame(inquiry_post1, inquiry_post2, inquiry_post3), na.rm=T)
         )

descr <- describeBy(pbl_di_desc, group = pbl_di_desc$treatment)

descr$`0`[2:10]
```

```
##                   n  mean    sd median trimmed  mad  min     max   range
## treatment       306  0.00  0.00   0.00    0.00 0.00 0.00    0.00    0.00
## attendance      220  1.81  0.41   2.00    1.89 0.00 0.00    2.00    2.00
## sel_att_pre1    293  2.54  2.06   2.00    2.19 1.48 0.00   12.00   12.00
## sel_att_pre2    293  2.32  1.93   2.00    2.00 1.48 0.00   14.00   14.00
## sel_att_pre3    293  2.46  2.22   1.00    2.10 1.48 0.00   17.00   17.00
## sel_att_post1   230  2.31  1.89   1.50    2.03 0.74 0.00    9.00    9.00
## sel_att_post2   230  2.38  1.89   2.00    2.05 1.48 0.00   12.00   12.00
## sel_att_post3   230  1.87  1.42   1.00    1.65 0.00 0.00    8.00    8.00
## theory_pre1     285  0.07  0.24   0.00    0.00 0.00 0.00    1.00    1.00
## theory_pre2     281  0.05  0.19   0.00    0.00 0.00 0.00    1.00    1.00
## theory_pre3     280  0.04  0.19   0.00    0.00 0.00 0.00    1.00    1.00
## theory_post1    215  0.27  0.40   0.00    0.22 0.00 0.00    1.00    1.00
## theory_post2    225  0.23  0.38   0.00    0.17 0.00 0.00    1.00    1.00
## theory_post3    217  0.19  0.36   0.00    0.12 0.00 0.00    1.00    1.00
## inquiry_pre1    285  2.36  0.62   2.50    2.43 0.74 1.00    3.00    2.00
## inquiry_pre2    281  2.09  0.70   2.00    2.12 0.74 0.67    3.00    2.33
## inquiry_pre3    280  2.10  0.76   2.00    2.13 1.15 0.00    3.00    3.00
## inquiry_post1   215  2.30  0.65   2.14    2.36 0.95 0.67    3.00    2.33
## inquiry_post2   225  2.06  0.70   2.00    2.07 0.89 1.00    3.00    2.00
## inquiry_post3   217  2.10  0.73   2.00    2.12 0.99 0.67    3.00    2.33
## literature      220  1.19  1.19   1.00    1.11 1.48 0.00    3.00    3.00
## doz_pass        306  3.97  1.76   3.00    3.96 1.48 2.00    6.00    4.00
## doz_gef         306  3.61  1.92   3.00    3.63 2.97 1.00    6.00    5.00
## geschl          302  1.33  0.48   1.00    1.28 0.00 1.00    3.00    2.00
## unterrichtet    300 12.81 91.04   2.00    3.01 2.97 0.00 1500.00 1500.00
## erf_vid         281  2.56  2.36   2.00    2.17 1.48 0.00   22.00   22.00
## lit_pre         286  0.47  0.81   0.00    0.29 0.00 0.00    3.00    3.00
## prior_knowledge 274  3.66  0.85   3.71    3.70 0.74 1.21    6.00    4.79
## sel_att_pre     293  2.44  1.71   2.00    2.19 1.48 0.33   14.33   14.00
## sel_att_post    230  2.19  1.41   1.67    1.97 0.99 0.33    7.33    7.00
## theory_pre      293  0.05  0.14   0.00    0.01 0.00 0.00    1.00    1.00
## theory_post     230  0.23  0.33   0.06    0.17 0.09 0.00    1.00    1.00
## inquiry_pre     293  2.18  0.58   2.17    2.20 0.74 1.00    3.00    2.00
## inquiry_post    230  2.16  0.59   2.17    2.17 0.74 0.78    3.00    2.22
```

**Group 1: PBL**

```
descr$`1`[2:10]
```

```
##                   n  mean     sd median trimmed  mad  min     max   range
## treatment       332  1.00   0.00   1.00    1.00 0.00 1.00    1.00    0.00
## attendance      247  1.74   0.47   2.00    1.82 0.00 0.00    2.00    2.00
## sel_att_pre1    323  2.60   2.18   2.00    2.25 1.48 0.00   18.00   18.00
## sel_att_pre2    323  2.23   2.07   1.00    1.83 1.48 0.00   12.00   12.00
## sel_att_pre3    323  2.42   2.33   2.00    2.06 1.48 0.00   15.00   15.00
## sel_att_post1   245  2.24   1.86   2.00    1.95 1.48 0.00   12.00   12.00
## sel_att_post2   245  2.39   2.03   2.00    2.09 1.48 0.00   13.00   13.00
## sel_att_post3   245  1.82   1.47   1.00    1.63 0.00 0.00   10.00   10.00
## theory_pre1     314  0.08   0.25   0.00    0.00 0.00 0.00    1.00    1.00
## theory_pre2     308  0.02   0.13   0.00    0.00 0.00 0.00    1.00    1.00
## theory_pre3     291  0.03   0.13   0.00    0.00 0.00 0.00    1.00    1.00
## theory_post1    229  0.31   0.42   0.00    0.27 0.00 0.00    1.00    1.00
## theory_post2    226  0.31   0.41   0.00    0.26 0.00 0.00    1.00    1.00
## theory_post3    225  0.22   0.39   0.00    0.16 0.00 0.00    1.00    1.00
## inquiry_pre1    313  2.30   0.66   2.33    2.38 0.99 0.00    3.00    3.00
## inquiry_pre2    308  2.16   0.73   2.00    2.21 0.99 0.00    3.00    3.00
## inquiry_pre3    291  2.04   0.71   2.00    2.05 0.89 0.00    3.00    3.00
## inquiry_post1   229  2.35   0.66   2.50    2.43 0.74 0.00    3.00    3.00
## inquiry_post2   225  2.13   0.67   2.00    2.17 0.74 0.00    3.00    3.00
## inquiry_post3   225  2.16   0.76   2.00    2.20 1.48 0.00    3.00    3.00
## literature      247  1.36   1.31   1.00    1.33 1.48 0.00    3.00    3.00
## doz_pass        332  4.94   1.37   6.00    5.16 0.00 2.00    6.00    4.00
## doz_gef         332  4.64   1.37   5.00    4.79 1.48 2.00    6.00    4.00
## geschl          332  1.35   0.48   1.00    1.32 0.00 1.00    2.00    1.00
## unterrichtet    327 34.08 497.67   2.00    2.94 2.97 0.00 9000.00 9000.00
## erf_vid         305  2.65   2.72   2.00    2.20 1.48 0.00   30.00   30.00
## lit_pre         313  0.52   0.92   0.00    0.30 0.00 0.00    3.00    3.00
## prior_knowledge 303  3.69   0.86   3.79    3.72 0.74 1.00    6.00    5.00
## sel_att_pre     323  2.42   1.81   2.00    2.13 1.48 0.33   14.67   14.33
## sel_att_post    245  2.15   1.44   1.67    1.94 0.99 0.33    9.00    8.67
## theory_pre      323  0.04   0.11   0.00    0.01 0.00 0.00    0.67    0.67
## theory_post     245  0.27   0.34   0.08    0.22 0.12 0.00    1.00    1.00
## inquiry_pre     323  2.17   0.56   2.17    2.19 0.62 0.00    3.00    3.00
## inquiry_post    245  2.22   0.60   2.28    2.26 0.66 0.00    3.00    3.00
```

**Descriptive Plot 1**  
Differentiated by treatment and vignettes of pre and post test.

```
# pivot data for plot
pbl_di_p <- pbl_di %>%
  dplyr::mutate(id = 1:length(sel_att_pre1)) %>%   # create id variable
  dplyr::select(id,treatment,
         sel_att_pre1, sel_att_pre2, sel_att_pre3, sel_att_post1, sel_att_post2, sel_att_post3,
         theory_pre1, theory_pre2, theory_pre3, theory_post1, theory_post2, theory_post3, 
         inquiry_pre1, inquiry_pre2, inquiry_pre3, inquiry_post1, inquiry_post2, inquiry_post3) %>%
  pivot_longer(cols = c(3:20),                                         # pivot longer first
               names_to = "variables",
               values_to = "values",
               values_transform = as.numeric) %>%
  dplyr::mutate(vignette = case_when(                                  # separate vignette number from variable name
                                variables == "sel_att_pre1" ~ "pre_1", 
                                variables == "sel_att_pre2" ~ "pre_2", 
                                variables == "sel_att_pre3" ~ "pre_3", 
                                variables == "sel_att_post1" ~ "post_1", 
                                variables == "sel_att_post2" ~ "post_2", 
                                variables == "sel_att_post3" ~ "post_3",
                                variables == "theory_pre1" ~ "pre_1", 
                                variables == "theory_pre2" ~ "pre_2", 
                                variables == "theory_pre3" ~ "pre_3", 
                                variables == "theory_post1" ~ "post_1", 
                                variables == "theory_post2" ~ "post_2", 
                                variables == "theory_post3" ~ "post_3",
                                variables == "inquiry_pre1" ~ "pre_1", 
                                variables == "inquiry_pre2" ~ "pre_2", 
                                variables == "inquiry_pre3" ~ "pre_3", 
                                variables == "inquiry_post1" ~ "post_1", 
                                variables == "inquiry_post2" ~ "post_2", 
                                variables == "inquiry_post3" ~ "post_3"),
                vignette = factor(vignette,
                                  levels = c("pre_1", "pre_2", "pre_3", "post_1", "post_2", "post_3")),
                variables = case_when(                                      # shorten variable names
                                variables == "sel_att_pre1" ~ "selection", 
                                variables == "sel_att_pre2" ~ "selection", 
                                variables == "sel_att_pre3" ~ "selection", 
                                variables == "sel_att_post1" ~ "selection", 
                                variables == "sel_att_post2" ~ "selection", 
                                variables == "sel_att_post3" ~ "selection",
                                variables == "theory_pre1" ~ "theory", 
                                variables == "theory_pre2" ~ "theory", 
                                variables == "theory_pre3" ~ "theory", 
                                variables == "theory_post1" ~ "theory", 
                                variables == "theory_post2" ~ "theory", 
                                variables == "theory_post3" ~ "theory",
                                variables == "inquiry_pre1" ~ "inquiry", 
                                variables == "inquiry_pre2" ~ "inquiry", 
                                variables == "inquiry_pre3" ~ "inquiry", 
                                variables == "inquiry_post1" ~ "inquiry", 
                                variables == "inquiry_post2" ~ "inquiry", 
                                variables == "inquiry_post3" ~ "inquiry")
                ) %>%
  pivot_wider(id_cols = c("id", "vignette", "treatment"),                                # then pivot back wide
              names_from = "variables", 
              values_from = "values") %>%
  dplyr::filter(!is.na(theory) | !is.na(inquiry)) %>%
  group_by(vignette, treatment) %>%
  dplyr::summarize(selection = mean(selection, na.rm = T),
                   inquiry = mean(inquiry, na.rm = T),
                   theory = mean(theory, na.rm = T)) %>%
  dplyr::mutate(prepost = case_when(vignette == "pre_1" | vignette == "pre_2" | vignette == "pre_3" ~ "pre",
                                    vignette == "post_1" | vignette == "post_2" | vignette == "post_3" ~ "post")) %>%
  ungroup()


p <-
ggplot(pbl_di_p, aes(x = inquiry, y = theory)) +
  # stat_density_2d(aes(fill = stat(level)), geom = "polygon", alpha = .3, h = c(1.2,1.2), show.legend = F) +
  # scale_fill_viridis_c() +
  geom_point(aes(color=vignette, shape = factor(treatment, labels = c("DI", "PBL"))), size = 4) +
  theme_few() +
  scale_shape_manual(values = c(12,15)) +
  scale_color_manual(values = c("#00B6D6", "#0B51E3", "#0AD182",
                               "#D60E00", "#D10A9E", "#FF620D")) +
  scale_fill_manual(values = c("#00B6D6", "#0B51E3", "#0AD182",
                               "#D60E00", "#D10A9E", "#FF620D")) +
  scale_y_continuous(limits = c(0,.5), breaks = c(0,.1,.2,.3,.4,.5), minor_breaks = NULL, expand = c(0, 0)) +
  scale_x_continuous(limits = c(0,3), breaks = c(0,1,2,3), minor_breaks = NULL, expand = c(0, 0)) +
  xlab("Realized inquiry steps in analyses") +
  ylab("% of analyses containing theory-practice integration")
  
p$labels$shape <- "treatment"
# ggsave("Fig2.tiff", width = 90, height = 70, units = "mm", dpi = 500, scale = 1.6)

p
```

**Descriptive Plot 2**  
Change from pretest to posttest on realizes inquiry steps and theory-practice integration differentiated by treatment.

```
pbl_di_p2 <- pbl_di %>%
  mutate(id = 1:nrow(.),
         theory_pre1 = as.numeric(theory_pre1),
         theory_pre2 = as.numeric(theory_pre2),
         theory_pre3 = as.numeric(theory_pre3),
         theory_post1 = as.numeric(theory_post1),
         theory_post2 = as.numeric(theory_post2),
         theory_post3 = as.numeric(theory_post3),
         theory_pre = rowMeans(data.frame(theory_pre1, theory_pre2, theory_pre3)),
         theory_pos = rowMeans(data.frame(theory_post1, theory_post2, theory_post3)),
         inquir_pre = rowMeans(data.frame(inquiry_pre1, inquiry_pre2, inquiry_pre3)),
         inquir_pos = rowMeans(data.frame(inquiry_post1, inquiry_post2, inquiry_post3)),
         theory = theory_pos - theory_pre,
         inquiry = inquir_pos - inquir_pre) %>%
  dplyr::select(id, treatment, theory, inquiry)


pbl_di_p2$treatment <- factor(pbl_di_p2$treatment, 
                              levels = c(0,1), 
                              labels = c("DI", "PB"))


ggplot(pbl_di_p2, aes(x = inquiry, y = theory)) +
  stat_density_2d(aes(fill = ..level..), geom = "polygon")  +
  scale_fill_continuous(type = "viridis") +
  scale_y_continuous(limits = c(-1.1,1.1), labels = c("-100%", "-50%", "0%", "+50%", "+100%"), 
                     minor_breaks = NULL, expand = c(0, 0)) +
  scale_x_continuous(limits = c(-3.1,3.1), breaks = c(-3,-2,-1,0,1,2,3), 
                     labels = c("-3", "-2", "-1", "0", "+1", "+2", "+3"), 
                     minor_breaks = NULL, expand = c(0, 0)) +
  xlab("change in realized inquiry steps in analyses") +
  ylab("change in % of analyses \ncontaining theory-practice integration") +
  labs(fill = "density") +
  geom_hline(yintercept=0, color = "white", alpha = .2) +
  geom_vline(xintercept=0, color = "white", alpha = .2) +
  theme_bw() +
  facet_wrap(~treatment)
```

**Descriptive Plot 3**  
Change from pretest to posttest in theory-practice integrations plotted with the instructor’s positive attitude.

```
pbl_di_p3 <- pbl_di %>%
  mutate(doz = rowMeans(data.frame(doz_pass, doz_gef), na.rm = T),
         theory_pre = rowMeans(data.frame(theory_pre1, theory_pre2, theory_pre3), na.rm = T),
         theory_post = rowMeans(data.frame(theory_post1, theory_post2, theory_post3), na.rm = T),
         theory_change = theory_post - theory_pre) %>%
  dplyr::select(doz, treatment, theory_pre, theory_post, theory_change) %>%
  pivot_longer(cols=3:5, names_to = "time", values_to = "theory")


ggplot(pbl_di_p3%>%dplyr::filter(time=="theory_change"), aes(x=doz, y=theory)) +
  stat_density_2d(aes(fill = ..level..), geom = "polygon")  +
  geom_smooth(method='lm', color = "red") +
  scale_fill_continuous(type = "viridis") +
  scale_y_continuous(limits = c(-.5,1.2), 
                     expand = c(0,0), 
                     breaks = c(-.4, -.2, 0,.2,.4,.6,.8,1),
                     labels = c("-40%", "-20%", "0%", "+20%", "+40%", "+60%", "+80%", "+100%")
                     ) +
  scale_x_continuous(limits = c(0.5,7), 
                     expand = c(0,0), 
                     breaks = c(1:6)) +
  geom_xsidedensity(aes(y=stat(density)), fill = "#37678c", color = NA) +
  geom_ysidedensity(aes(x=stat(density)), fill = "#37678c", color = NA, scale = "free_y") +
  scale_ysidex_continuous() +
  scale_xsidey_continuous() +
  xlab("instructor's positive attitude") +
  ylab("change in % of analyses \ncontaining theory-practice integration") +
  labs(fill = "density") +
  theme_bw()
```

## Inference Statistics

Based on imputed data.

### Hypothesis 1: DV ‘selection’

```
mulest <- matrix(0,
                 nrow=1000,
                 ncol=2
                 )
covwithin <- matrix(0,
                    nrow=2,
                    ncol=2
                    )

# execute 1000 multiple regressions using the imputed data matrices and store the estimates 
# of only the regression coefficients of funumb on prenumb and postnumband and the average 
# of the 1000 covariance matrices.
# See Hoijtink, Gu, Mulder, and Rosseel (2019) for an explanation of the latter.
model <- '
          select_t1 =~ sel_att_pre1 + sel_att_pre2 + sel_att_pre3
  
          select_t2 =~ sel_att_post1 + sel_att_post2 + sel_att_post3
  
          instr_beliefs =~ lamda*doz_pass + lamda*doz_gef
          
          select_t2 ~ select_t1 + treatment + instr_beliefs + literature + attendance
         '

fit_mean <- data.frame(chi_sq = c(),
                       chi_df = c(),
                       chi_p = c(),
                       cfi_robust = c(),
                       tli_robust = c(),
                       rmsea_robust = c(),
                       rmsea_cilower_robust = c(),
                       rmsea_ciupper_robust = c())

for(i in 1:M) {
  # compute model
  fit <- sem(model = model,
             data = complete(imp,i),
             cluster = "seminar",
             se = "robust",
             orthogonal = T,
             std.lv = T
             )
  
  # save coefficients
  mulest[i,]<-coef(fit)[10:11]
  covwithin<-covwithin + 1/M * vcov(fit)[10:11,10:11]
  
  # save fit indices
  fit_mean[i, "chi_sq"] <- fitMeasures(fit)[6]
  fit_mean[i, "chi_df"] <- fitMeasures(fit)[7]
  fit_mean[i, "chi_p"] <- fitMeasures(fit)[8]
  fit_mean[i, "cfi_robust"] <- fitMeasures(fit)[27]
  fit_mean[i, "tli_robust"] <- fitMeasures(fit)[28]
  fit_mean[i, "rmsea_robust"] <- fitMeasures(fit)[48]
  fit_mean[i, "rmsea_cilower_robust"] <- fitMeasures(fit)[49]
  fit_mean[i, "rmsea_ciupper_robust"] <- fitMeasures(fit)[50]
}


# average fit indices
fit_mean <- fit_mean %>%
  dplyr::summarize_all(mean)

fit_mean
```

```
##     chi_sq chi_df      chi_p cfi_robust tli_robust rmsea_robust
## 1 56.61463     40 0.05305228  0.9811482  0.9754927   0.02532669
##   rmsea_cilower_robust rmsea_ciupper_robust
## 1           0.01433763           0.03445862
```

```
# Compute the average of the estimates and assign names, the between and total covariance matrix.
# See Hoijtink, Gu, Mulder, and Rosseel (2019) for an explanation.
estimates <- colMeans(mulest)
names(estimates) <- c("treatment", "instr_beliefs")
covbetween <- cov(mulest)
covariance <- covwithin + (1+1/M)*covbetween

# determine the sample size
samp <- nrow(pbl_di)

# compute the effective sample size
# See Hoijtink, Gu, Mulder, and Rosseel (2019) for an explanation.
nucom<-samp-length(estimates)
lam <- (1+1/M)*(1/length(estimates))* sum(diag(covbetween %*% ginv(covariance)))
nuold<-(M-1)/(lam^2)
nuobs<-(nucom+1)/(nucom+3)*nucom*(1-lam)
nu<- nuold*nuobs/(nuold+nuobs)
fracmis <- (nu+1)/(nu+3)*lam + 2/(nu+3)
neff<-samp-samp*fracmis
covariance<-list(covariance)

# set the seed
set.seed(100)
# test hypotheses with bain
results <- bain(estimates,
                "instr_beliefs>0 & treatment=0;
                instr_beliefs<0 & treatment=0;
                instr_beliefs=0 & treatment=0",
                n=neff,
                Sigma=covariance,
                group_parameters=2,
                joint_parameters = 0)
# display the results
print(results)
```

```
## Bayesian informative hypothesis testing for an object of class numeric:
## 
##    Fit   Com   BF.u   BF.c   PMPa  PMPb  PMPc 
## H1 0.050 0.097 0.518  0.518  0.010 0.010 0.010
## H2 2.619 0.097 27.116 27.116 0.516 0.506 0.506
## H3 1.844 0.074 24.944 24.944 0.474 0.466 0.466
## Hu                                 0.019      
## Hc                                       0.019
## 
## Hypotheses:
##   H1: instr_beliefs>0&treatment=0
##   H2: instr_beliefs<0&treatment=0
##   H3: instr_beliefs=0&treatment=0
## 
## Note: BF.u denotes the Bayes factor of the hypothesis at hand versus the unconstrained hypothesis Hu. BF.c denotes the Bayes factor of the hypothesis at hand versus its complement. PMPa contains the posterior model probabilities of the hypotheses specified. PMPb adds Hu, the unconstrained hypothesis. PMPc adds Hc, the complement of the union of the hypotheses specified.
```

```
# display BFs of Hypotheses against each other
results$BFmatrix
```

```
##          H1         H2         H3
## H1  1.00000 0.01908576 0.02074778
## H2 52.39510 1.00000000 1.08708165
## H3 48.19794 0.91989410 1.00000000
```

### Hypothesis 2: DV ‘inquiry’

```
mulest <- matrix(0,
                 nrow=1000,
                 ncol=2
                 )
covwithin <- matrix(0,
                    nrow=2,
                    ncol=2
                    )

# execute 1000 multiple regressions using the imputed data matrices and store the estimates 
# of only the regression coefficients of funumb on prenumb and postnumband and the average 
# of the 1000 covariance matrices.
# See Hoijtink, Gu, Mulder, and Rosseel (2019) for an explanation of the latter.
model <- '
          inquiry_t1 =~ inquiry_pre1 + inquiry_pre2 + inquiry_pre3
  
          inquiry_t2 =~ inquiry_post1 + inquiry_post2 + inquiry_post3
  
          instr_beliefs =~ lamda*doz_pass + lamda*doz_gef
          
          inquiry_t2 ~ inquiry_t1 + treatment + instr_beliefs + literature + attendance
         '

fit_mean <- data.frame(chi_sq = c(),
                       chi_df = c(),
                       chi_p = c(),
                       cfi_robust = c(),
                       tli_robust = c(),
                       rmsea_robust = c(),
                       rmsea_cilower_robust = c(),
                       rmsea_ciupper_robust = c())

for(i in 1:M) {
  # compute model
  fit <- sem(model = model,
             data = complete(imp,i),
             cluster = "seminar",
             se = "robust",
             orthogonal = T,
             std.lv = T
             )
  
  # save coefficients
  mulest[i,]<-coef(fit)[10:11]
  covwithin<-covwithin + 1/M * vcov(fit)[10:11,10:11]
  
  # save fit indices
  fit_mean[i, "chi_sq"] <- fitMeasures(fit)[6]
  fit_mean[i, "chi_df"] <- fitMeasures(fit)[7]
  fit_mean[i, "chi_p"] <- fitMeasures(fit)[8]
  fit_mean[i, "cfi_robust"] <- fitMeasures(fit)[27]
  fit_mean[i, "tli_robust"] <- fitMeasures(fit)[28]
  fit_mean[i, "rmsea_robust"] <- fitMeasures(fit)[48]
  fit_mean[i, "rmsea_cilower_robust"] <- fitMeasures(fit)[49]
  fit_mean[i, "rmsea_ciupper_robust"] <- fitMeasures(fit)[50]
}


# average fit indices
fit_mean <- fit_mean %>%
  dplyr::summarize_all(mean)

fit_mean
```

```
##     chi_sq chi_df      chi_p cfi_robust tli_robust rmsea_robust
## 1 56.58565     40 0.05916684  0.9811833  0.9755382   0.02518879
##   rmsea_cilower_robust rmsea_ciupper_robust
## 1            0.0133403           0.03477881
```

```
# Compute the average of the estimates and assign names, the between and total covariance matrix.
# See Hoijtink, Gu, Mulder, and Rosseel (2019) for an explanation.
estimates <- colMeans(mulest)
names(estimates) <- c("treatment", "instr_beliefs")
covbetween <- cov(mulest)
covariance <- covwithin + (1+1/M)*covbetween

# determine the sample size
samp <- nrow(pbl_di)

# compute the effective sample size
# See Hoijtink, Gu, Mulder, and Rosseel (2019) for an explanation.
nucom<-samp-length(estimates)
lam <- (1+1/M)*(1/length(estimates))* sum(diag(covbetween %*% ginv(covariance)))
nuold<-(M-1)/(lam^2)
nuobs<-(nucom+1)/(nucom+3)*nucom*(1-lam)
nu<- nuold*nuobs/(nuold+nuobs)
fracmis <- (nu+1)/(nu+3)*lam + 2/(nu+3)
neff<-samp-samp*fracmis
covariance<-list(covariance)

# set the seed
set.seed(100)
# test hypotheses with bain
results <- bain(estimates,
                "treatment>0 & instr_beliefs>0;
                treatment=0 & instr_beliefs>0;
                treatment>0 & instr_beliefs=0;
                treatment=0 & instr_beliefs=0;",
                n=neff,
                Sigma=covariance,
                group_parameters=2,
                joint_parameters = 0)
# display the results
print(results)
```

```
## Bayesian informative hypothesis testing for an object of class numeric:
## 
##    Fit   Com   BF.u   BF.c   PMPa  PMPb  PMPc 
## H1 0.681 0.233 2.927  7.038  0.025 0.024 0.025
## H2 1.803 0.089 20.161 20.161 0.169 0.168 0.169
## H3 2.662 0.197 13.481 13.481 0.113 0.112 0.113
## H4 5.862 0.071 82.456 82.456 0.693 0.687 0.690
## Hu                                 0.008      
## Hc 0.319 0.767 0.416                     0.003
## 
## Hypotheses:
##   H1: treatment>0&instr_beliefs>0
##   H2: treatment=0&instr_beliefs>0
##   H3: treatment>0&instr_beliefs=0
##   H4: treatment=0&instr_beliefs=0
## 
## Note: BF.u denotes the Bayes factor of the hypothesis at hand versus the unconstrained hypothesis Hu. BF.c denotes the Bayes factor of the hypothesis at hand versus its complement. PMPa contains the posterior model probabilities of the hypotheses specified. PMPb adds Hu, the unconstrained hypothesis. PMPc adds Hc, the complement of the union of the hypotheses specified.
```

```
# display BFs of Hypotheses against each other
results$BFmatrix
```

```
##           H1        H2        H3         H4
## H1  1.000000 0.1451863 0.2171192 0.03549816
## H2  6.887703 1.0000000 1.4954523 0.24450077
## H3  4.605766 0.6686940 1.0000000 0.16349620
## H4 28.170476 4.0899666 6.1163499 1.00000000
```

### Hypothesis 3: DV ‘theory’

```
mulest <- matrix(0,
                 nrow=1000,
                 ncol=2
                 )
covwithin <- matrix(0,
                    nrow=2,
                    ncol=2
                    )

# execute 1000 multiple regressions using the imputed data matrices and store the estimates 
# of only the regression coefficients of funumb on prenumb and postnumband and the average 
# of the 1000 covariance matrices.
# See Hoijtink, Gu, Mulder, and Rosseel (2019) for an explanation of the latter.
model <- '
          theory_t1 =~ theory_pre1 + theory_pre2 + theory_pre3
  
          theory_t2 =~ theory_post1 + theory_post2 + theory_post3
  
          instr_beliefs =~ lamda*doz_pass + lamda*doz_gef
          
          theory_t2 ~ theory_t1 + treatment + instr_beliefs + literature + attendance
         '

fit_mean <- data.frame(chi_sq = c(),
                       chi_df = c(),
                       chi_p = c(),
                       cfi_robust = c(),
                       tli_robust = c(),
                       rmsea_robust = c(),
                       rmsea_cilower_robust = c(),
                       rmsea_ciupper_robust = c())

for(i in 1:M) {
  # compute model
  fit <- sem(model = model,
             data = complete(imp,i),
             cluster = "seminar",
             se = "robust",
             orthogonal = T,
             std.lv = T
             )
  
  # save coefficients
  mulest[i,]<-coef(fit)[10:11]
  covwithin<-covwithin + 1/M * vcov(fit)[10:11,10:11]
  
  # save fit indices
  fit_mean[i, "chi_sq"] <- fitMeasures(fit)[6]
  fit_mean[i, "chi_df"] <- fitMeasures(fit)[7]
  fit_mean[i, "chi_p"] <- fitMeasures(fit)[8]
  fit_mean[i, "cfi_robust"] <- fitMeasures(fit)[27]
  fit_mean[i, "tli_robust"] <- fitMeasures(fit)[28]
  fit_mean[i, "rmsea_robust"] <- fitMeasures(fit)[48]
  fit_mean[i, "rmsea_cilower_robust"] <- fitMeasures(fit)[49]
  fit_mean[i, "rmsea_ciupper_robust"] <- fitMeasures(fit)[50]
}


# average fit indices
fit_mean <- fit_mean %>%
  dplyr::summarize_all(mean)

fit_mean
```

```
##     chi_sq chi_df       chi_p cfi_robust tli_robust rmsea_robust
## 1 67.71363     40 0.008857133  0.9620182  0.9506237   0.03274186
##   rmsea_cilower_robust rmsea_ciupper_robust
## 1           0.02356483           0.04140744
```

```
# Compute the average of the estimates and assign names, the between and total covariance matrix.
# See Hoijtink, Gu, Mulder, and Rosseel (2019) for an explanation.
estimates <- colMeans(mulest)
names(estimates) <- c("treatment", "instr_beliefs")
covbetween <- cov(mulest)
covariance <- covwithin + (1+1/M)*covbetween

# determine the sample size
samp <- nrow(pbl_di)

# compute the effective sample size
# See Hoijtink, Gu, Mulder, and Rosseel (2019) for an explanation.
nucom<-samp-length(estimates)
lam <- (1+1/M)*(1/length(estimates))* sum(diag(covbetween %*% ginv(covariance)))
nuold<-(M-1)/(lam^2)
nuobs<-(nucom+1)/(nucom+3)*nucom*(1-lam)
nu<- nuold*nuobs/(nuold+nuobs)
fracmis <- (nu+1)/(nu+3)*lam + 2/(nu+3)
neff<-samp-samp*fracmis
covariance<-list(covariance)

# set the seed
set.seed(100)
# test hypotheses with bain
results <- bain(estimates,
                "treatment>0 & instr_beliefs>0;
                treatment=0 & instr_beliefs>0;
                treatment>0 & instr_beliefs=0;
                treatment=0 & instr_beliefs=0;",
                n=neff,
                Sigma=covariance,
                group_parameters=2,
                joint_parameters = 0
                )
# display the results
print(results)
```

```
## Bayesian informative hypothesis testing for an object of class numeric:
## 
##    Fit   Com   BF.u   BF.c   PMPa  PMPb  PMPc 
## H1 0.464 0.167 2.786  4.334  0.073 0.071 0.072
## H2 1.804 0.053 34.068 34.068 0.894 0.871 0.879
## H3 0.045 0.117 0.384  0.384  0.010 0.010 0.010
## H4 0.025 0.029 0.877  0.877  0.023 0.022 0.023
## Hu                                 0.026      
## Hc 0.536 0.833 0.643                     0.017
## 
## Hypotheses:
##   H1: treatment>0&instr_beliefs>0
##   H2: treatment=0&instr_beliefs>0
##   H3: treatment>0&instr_beliefs=0
##   H4: treatment=0&instr_beliefs=0
## 
## Note: BF.u denotes the Bayes factor of the hypothesis at hand versus the unconstrained hypothesis Hu. BF.c denotes the Bayes factor of the hypothesis at hand versus its complement. PMPa contains the posterior model probabilities of the hypotheses specified. PMPb adds Hu, the unconstrained hypothesis. PMPc adds Hc, the complement of the union of the hypotheses specified.
```

```
# display BFs of Hypotheses against each other
results$BFmatrix
```

```
##            H1         H2        H3         H4
## H1  1.0000000 0.08179050  7.265685  3.1769039
## H2 12.2263593 1.00000000 88.832881 38.8419679
## H3  0.1376333 0.01125709  1.000000  0.4372476
## H4  0.3147719 0.02574535  2.287033  1.0000000
```
