# Supplementary material for: Using authentic representations of practice in teacher education: Do direct instructional and problem-based approaches really produce different effects?
Source: PLoS One. 2022 Sep 2;17(9):e0273988. doi: 10.1371/journal.pone.0273988 (PMC9439238; doi:10.1371/journal.pone.0273988)
Supplement: Attachment — Submitted filename: Response to Reviewers.pdf [file pone.0273988.s003.pdf]

Reviewer #1	Our Response
I also tried to reproduce your results but I was missing the data set "delete.Rdata". I liked that you provided a markdown file with all your code.	Thank you for bringing this problem to our attention. We unnecessarily blinded the document with the analyses. The link to both data sets is now in the RMarkdown file. We also put the "delete.RDdta" data set on github in the "data_public" folder and renamed it to "ts.Rdata". How to download the data can be found under the heading "Import data" in the RMarkdown file.
I think the summary of the reviews could be more concise, more to the point. There is no advantage of citing that researchers are "reinventing the wheel" (ll125f).	We agree that the literature review would benefit from being restructured and rewritten for clarity. Therefore, we restructured and rewrote the introductory section, synthesizing the results of the reviews and tailoring literature review to the research questions.<br><br>We have deleted the statement on researchers "reinventing the wheel".
You claim to compare Problem Based Learning and Direct Instruction and your description of the methods you used fits these terms. However, in my particular understanding the definition of DI from Kirschner (2006) that you bring, would only define a “darstellende Stoffvermittlung”. In the current agreement of educational science, Direct Instruction names a specific instruction method that includes multiple phases.	We agree that the definition by Kirschner (2006) is too narrow for our conceptualization of direct instruction. We changed this statement to:<br>“In contrast, DI describes a teacher-centered approach in which phases of modeling are typically followed by phases of guided and individual practice (Stockard et al., 2018).”
The hypotheses are clear and could be tackled with this data. It would help the understanding of the results, if you already here make clear that you always test the sub-hypotheses in combination (and give a reason for it).	We now included a statement below the description of hypotheses:<br>“We tested the hypotheses of the two predictors (instructional approaches, instructors’ attitudes) within each dependent variable simultaneously to increase rigor by making the predictions as precise as possible.”
You use the number of attended sessions, the number of articles read for these sessions and instructor’s attitude towards the instruction method as proxies/control variables. However, it remains unclear, what these proxies are really measuring. A more thorough discussion would be indicated.	We agree that a more thorough discussion of these measures would be indicated. Therefore, we added paragraphs on the social desirability and interpretation of these measures in the sections 2.4.4, 2.4.5 and 2.4.6.
From a pedagogical point of view I do not really find the task described in lines 329-331 as promoting learning. A good task (Arbeitsauftrag) should be structured more and be more elaborate.	We explicitly refrained from strongly structuring the task, especially against the background of the PB instruction. The instructors implemented the task in the sense of their instructional approach.
I wondered whether each instructor had courses in both conditions. Please specify.	We agree that this information was missing in the manuscript. We now added a sentence to the “Design” section:<br>“Each instructor taught DI an PB courses, the conditions were balanced within the instructors (teaching the same amount of both conditions, except when teaching an odd number of courses).”

In section 3.4.1. there appeared again a short literature overview. I would shift this to the introduction above.	We agree, thank you for bringing this to our attention. These contents have now been moved to the literature review section and integrated accordingly.
In section 3.4.2 I wondered why you tested for unidimensionality of the vignettes? Do you mean the raters score of reflective thought per vignette? Why did you only test for unidimensionality of reflective thought and not of selective attention as well?	We clarified this section by changing one sentence to “The raters’ scores were tested for one-dimensionality per vignette”. When coding qualitative data, it is useful to check for dimensionality. One-dimensionality may indicate that the coding does not covary with other factors (e.g., the part of the vignette to which the analysis being coded refers).<br><br>Selective attention was not tested for one-dimensionality as the score comprises the sum (count) of analyses regarding classroom management. Therefore, a calculation of the dimensionality is not possible. Theory–practice integration was not tested for one-dimensionality for the same reason.
When you report interrater reliability, please also specify how many cases have been rated by two (or even three) raters. Please also state what you did in cases in which the raters had different scores.	We agree that this is essential information for assessing interrater reliability. We have updated the sentence accordingly:<br>“Inter-rater reliabilities for all codings were computed based on randomly selected 20% of the approximately 7 600 comments written by the participants in the pretest and posttest. Cohen’s Kappa scores of the two trained raters were satisfactory (κ = .64–.77) and disagreements were resolved through discussion.”
When reading the sentence in ll. 395 it was unclear to me what you meant by it. After reading part 3.5, I understood that you aimed at matching similar vignettes to pre- and posttest. Please clarify.	We concede that the description might have been unclear. We restructured this section and added the sentence:<br>“We investigated the extent to which pairs of similar vignettes could be found from a classroom video, each of which was then split between the pretest and the posttest.”
Please make clearer, that you test the two sub-hypotheses of each dependent variable in combination. Currently, the indices of the hypotheses do not match and it is difficult to match the hypotheses and results. I would number the three main hypotheses with numbers from 1-3 and the different cases that you test (hypothesized direction, opposite direction, null hypothesis, unrestricted hypothesis) with letters from a-d for example.	We agree that the consistency can be increased by numbering the hypotheses and sub-hypotheses. Numbers and indices of hypotheses in the section “Research Questions and Hypotheses” now match those from the “Results” section. Further, we numbered hypotheses on selective attention as H1 ($H1_1$, $H1_2$, $H1_0$, $H1_u$), hypotheses on reflective thought as H2 ($H2_1$, $H2_2$, $H2_3$, $H2_0$, $H2_u$) and hypotheses on theory-practice integration as H3 ($H3_1$, $H3_2$, $H3_3$, $H3_0$, $H3_u$). The hypotheses we formulated in the section " Research Questions and Hypotheses" are each assigned the index 1 ($H1_1$, $H2_1$, $H3_1$).<br><br>Also, we now mention that the sub-hypotheses are tested simultaneously in the sections “Research Questions and Hypotheses” as well as in “Statistical Analyses”.
Figure 2: Would it be possible to depict the raw data instead of the 12 data points here? It is not really interesting how the theory-practice integration differs by the	We assume you refer to Fig. 3: A similar graph with raw data would be an interesting option, but is unfortunately very cluttered. Therefore, we calculated the

vignettes, but more how it differs by DI vs PBL and how strongly students vary in that. Raw data per student would be very interesting.	change scores for each person on the two variables and created a two-dimensional density plot of change, differentiated for treatment groups. We updated the figure and caption accordingly.
You state that you included the students' willingness for effort and their attitude on readiness for reflection, but I did not see any results regarding this. Is this reported in the supplementary material.	In the supplementary material we only included analyses directly mirrored in the manuscript. The inclusion of further exploratory analyses would overload the document.
You state "Students' theory–practice integrations when analyzing classroom situations greatly increased from pretest to posttest." Beforehand you ask the reader to be careful when comparing the scores between pre- and posttest, as the vignettes differ. I thus find that this conclusion is a bit far fetched. It could just be due to the different vignettes.	We agree that this result should be interpreted with greater caution. We rewrote the sentence as follows:<br>"Students' theory-practice integration scores in analyzing classroom situations improved greatly from the pretest to the posttest. However, this result should be taken with a grain of salt because the pretest and posttest are not equivalent, even though we matched them with great effort."
I very much liked that the supplementary material contains a knitted R markdown file containing all the code and results. I wondered why the authors put the data on the gesis server in a proprietary .sav format (maybe add a .txt or .csv file as well). Please also upload the data "delete.Rdata", as this is vital to reproduce your code.	We agree that a proprietary file format (such as .sav) is less accessible than open file formats (such as .Rdata). At the recommendation of GESIS, we uploaded a SAV-file (and not a CSV-file) to the repository. As opposed to CSV, SAV-files include the item labels and levels – but so does the .Rdata file format. This is why we also uploaded the data sets as .Rdata. The "delete.RDdta" data set is now on github in the "data_public" folder and we renamed it to "ts.Rdata". A guide on how to download the data can be found under the heading "Import data" in the RMarkdown file. The links to both relevant data sets are now in the RMarkdown file.
I suggest a sound proofreading focusing of the clarity and precision of the language. Also please make use of the past tense, and use it consistently. You may also consider asking the writing experts at your institution. From my experience, this is always greatly improving a manuscript.	We have thoroughly proofread the manuscript and revised it accordingly. For this we also involved an external writing expert.
Use key nouns consistently: for example, the manuscript uses "DI and PB methods"/"PB instruction"/"PB learning"/"PBL" → choose one. This also happens for "subject"/"student"/"teacher student" and "count"/"number"/"quantity" and "second semester"/"2nd semester"/"teachers grade level (second)". Please be very precise and consistent in the words you use.	We agree that consistency will foster clarity of the manuscript.<br>We decided for<br>• "DI/PB approach"<br>• "student" (however, we kept "student teachers" when it contributed to understanding)<br>• "number of selected situations"<br>• "second semester"<br>and updated the manuscript accordingly.
I would not number the overview of current research as "2.", but put it as sub-sections of the Introduction.	We updated the manuscript accordingly.

Reviewer #2	Our Response
The authors may need to restructure the manuscript and add a literature review on PBL and DI in teacher education. For example, the definition of PBL. What does other research already know about PBL and DI?<br>Why are the skills of selective attention, reflective thought and theory-practice integration important to students in teacher education? Are they difficult to be developed? Why do the authors anticipate PBL or DI could help students develop those three skills.	We have rewritten the introductory section and added remarks on the relevance of reflection and related constructs in teacher education.<br><br>For each section of selective attention, reflective thinking, and integration of theory and practice we elaborated their relevance to teacher professionalism and the development of professionalism (section “Selective attention, reflective thought and theory-practice integration with authentic representations of practice”).<br><br>Further you will now find theoretical and empirical elaborations on the efficacy of PBL and DI to develop these skills in the “Problem-based and direct instruction” section.
Moreover, some contents in the Method section should be moved to the literature review session. For instance, the first part of 3.4.1 (line 403 to 420) was the literature review about “Selective Attention”. Similarly, the first paragraph of 3.4.2, 3.4.3 and 3.4.4 should move to the literature review section.	We agree, thank you for bringing this to our attention. These contents have now been moved to the literature review section and integrated accordingly.
There are no research questions, only hypotheses in this study. However, the authors might need to provide the relational or evidence of the predictions based on previous research.	We now included research questions as well as hypotheses. Also, we have updated the introductory section to include literature that leads more directly to the predictions of the hypotheses.
The sentence in Line 314 is not clear.	We delted the sentence “In the two sessions, students learned about the classroom management strategies of Kounin (1970), Evertson (2006), and Mayr (2009).” and described the contents of the course in the design section.
LINE 281: What is the reason to redesign sessions 6 and 7, rather than other sessions?	We focused on redesigning sessions where classroom management was on the curriculum. This was the case for sessions six and seven.<br>We updated the sentences<br>“For the interventions, we redesigned two of the courses’ weekly 90-minute sessions (6th and 7th of 15 sessions) and an assignment between these two sessions using authentic representations of practice. The topic of these two sessions and the assignment was classroom management.”<br><br>to

	"For the interventions, we redesigned part of the courses (two sessions and an inter-session assignment) using authentic representations of practice. For this purpose, we focused on sessions in which classroom management was on the curriculum. These were sessions six and seven of 15 sessions."
Line 343: It is not clear that students analysed the situations in groups or individually.	Thank you for bringing this to our attention. We changed the sentence to "After this, students individually analyzed some more situations"
In Line 352, the authors mentioned that "To guide the analysis, students received key questions that targeted the analysis of practice steps". It means students were received the guidance of the analysis procedure, step by step. Based on one of the essential characteristics of PBL, " The problem simulations used in problem-based learning must be ill-structured and allow for free inquiry (p.13, Savery, J.R., 2006)". It seems the treatment in the PBL group was not ill-structured and didn't allow for free inquiry.	Our description in the manuscript may have been somewhat unclear:<br><br>The problem is ill-structured as students determine what they consider to be a problem (selection of situations) and what is and is not part of the problem.<br><br>The students were free to inquire solutions to these situations. The key questions merely served as a guide in case students needed support with their inquiry. The questions did not serve as step-by-step instructions and were not introduced as such. We now clarified this in the manuscript.
Line 379: Not sure where are the research questions of this study?	We now included research questions.
In section 4.1, I am keen to know why the number of analysed situations decreased in both groups.	This is indeed an interesting phenomenon that needs further investigation. However, our data unfortunately do not allow us to answer this question conclusively. We now offer several interpretations of this in the discussion section: „Furthermore, we found that the number of selected situations decreased from the pretest to the posttest in both conditions. We cannot conclusively elucidate this phenomenon with our data, but we offer some tentative interpretations. A first intuitive explanation is that the analyses became fewer because students wrote longer analyses. However, we did not observe an increase in the length of the analyses in our data. A second explanation could be that the vignettes to be analyzed in the pretest and the posttest offer different numbers of situations that can be analyzed. Although we matched the vignettes to the pretest and posttest with great effort, we cannot exclude this option. A third explanatory approach relates to test fatigue. The students may put more effort into the pretest because analyzing classroom videos was a novel format for them (novelty effect). After they went through the pretest and analyzed several instructional videos again in the treatment sessions, the novelty effect may have worn off and their willingness to reflect may have decreased in the posttest. A slight decrease in scales of readiness to reflect was indeed observed in our data. "

In section 4.2, the authors mentioned that "Students' reflective thought (as measured by realized inquiry steps in the analyses) was already well developed before they entered the treatment sessions". If this is the case, why did the authors measure their skills of reflective thoughts? The authors already know students have this skill before the intervention.	The time period that the students were given to complete the pretest extended until directly before the first treatment session. Accordingly, we were unfortunately not able to analyze the data before the treatment.
Line 607: The authors mentioned that " we conclude that the effect on students' reflective thought is equivalent between learning approaches". The authors had explained because students had already developed the skill of reflective thoughts before the interventions; therefore, there was no significant difference between the pre-and post-tests. Then the authors conclude that the effect on students' reflective thoughts is equivalent between learning approaches. Please indicate what evidence to make this conclusion.	The conclusion "that the effect on students' reflective thought is equivalent between learning approaches" is not related to our assumption that students had already developed the skill of reflective thoughts before the interventions. We derived this conclusion directly from our data: The inferential statistical comparison of the formulated hypotheses generated evidence for the null hypothesis. The null hypothesis states that there is no difference between the two groups. We therefore generated evidence that the effect is equivalent, which is possible with Bayes Factor hypothesis testing.
There is no discussion in the Discussion section.	We added a discussion on the decrease of the number of selected situations. Further, we added a paragraph on the relation of the analysis of practice and classroom practice itself.
