# Supplementary material for: Using authentic representations of practice in teacher education: Do direct instructional and problem-based approaches really produce different effects?
Source: PLoS One. 2022 Sep 2;17(9):e0273988. doi: 10.1371/journal.pone.0273988 (PMC9439238; doi:10.1371/journal.pone.0273988)
Supplement: Attachment — Submitted filename: Response to Reviewers.pdf [file pone.0273988.s004.pdf]

Reviewer comment:

First, the variable labels are unclear (doz_gef & doz_pass), and even lead to the suspicion, that these variables did not measure the attitude towards problem-based learning and direct instruction. Why did you choose these labels and which instructional approach do they refer to?

Our response:

“doz” is short for “Dozierende” which signifies “instructor” in German. As the data set consists mostly of data from students we wanted to make this clear in the variable label. The suffix “_gef” and “_pass” refers to the two items measuring their attitudes perceived “pleasure” and perceived “fit”.

Reviewer comment:

Secondly, the dataset "rating-treatmentcheck.sav" contains the real names of the instructors --> anonymise and give them a code or number.

Our response:

Thank you for this very important information. We followed your suggested procedure.

Reviewer comment:

Thirdly, and this is the major problem: the instructors were not consistent in their ratings about their attitudes. For example, the instructor of seminars 10, 11, and 12 gave the following answers on the attitudes: 3-4, 1-2, 4-4.

Our response:

I see how disagreement within instructors may seem like a substantial problem. However, the example you pointed out, come from within one instructor but between different treatments. Hence we would assume agreement (=similar ratings) within the same type of courses (=same treatment) but not necessarily between courses from different treatments within each instructor. This was the case here and is the case for all instructors. In the word document “Response to Reviewers” I have attached two figures showing the agreement/disagreement for the two items.

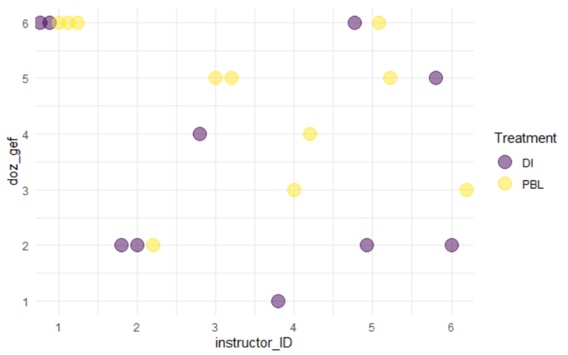

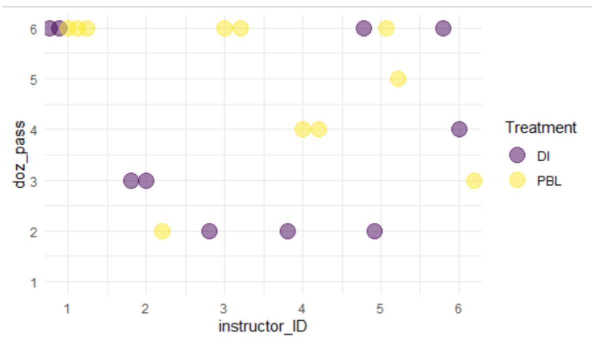

Reviewer comment:

line 698 ff. That is related to the instructors' attitudes again: I am not convinced by the argument that the broad range of attitudes indicates the absence of any bias of the instructors.

Our response:

Our wording "bias" might be somewhat unclear. We wanted to illustrate that attitudes varied considerably within the treatments signifying that each treatment did not experience "only disagree" or "only agree" and therefore instructors being one-sided throughout. We therefore changed the sentence to "This indicates that instructors had divergent but not necessarily one-sided attitudes throughout for each treatment, thus, we used the variable as a covariate of the treatment."

Reviewer comment:

Please state the mean attitude per treatment & SDs, and maybe consider a Bayesian t-test for dependent samples to check, whether the attitudes are comparable (after you resolved the issue that they vary across seminars).

Our response:

We did not expect instructors to be similar in their attitudes toward the treatment. For this reason, we included the variable as a predictor. The decision to be made was solely between using the variable as a moderator or as a covariate. We added the Bayes factor test and deleted the part on the mediation as it does not add to the manuscript and might be unclear.

Reviewer comment:

I am also not convinced to model these two distinct attitudes as one latent variable. It's not a truly reflective construct, is it? Wouldn't it be much more meaningful to see how attitudes towards DI predict theory-practice integration in the DI, whereas attitudes towards PB predict theory-practice integration in PB-approaches? Or maybe a difference score instead of a sum score? Not yet sure, but please explain in further detail, why you modeled the two attitudes as indicators of one underlying general attitude variable.

Our response:

Our description might have been somewhat unclear. First, the attitude toward the treatment was measured via two items. These two items both measure how positive the attitude of the instructors was toward a treatment. As instructors taught several courses, they indicated their attitude toward the treatment for each of the courses separately. In the data set then, the attitude of the instructor toward a specific course was matched to students' data from exactly that course. This way, we achieved exactly what you were suggesting: "attitudes towards DI predict theory-practice integration in the DI, whereas attitudes towards PB predict theory-practice integration in PB-approaches". To clarify this, we added the following paragraph: "We matched the attitude of the instructor toward a specific course to students' data from exactly that course. This way we were able to predict student level data with the respective course level information."

Reviewer comment:

A second major aspect concerns Figure 3. To me, Fig 3 makes no sense; in the hypothesis you do not test whether theory-practice integration relate to realized inquiry steps, so why plot it? For which research question does it depict meaningful information? I would put instructors' attitude on the x-axis and then add a Figure 4 to plot the change in theory-practice integration (again predicted by instructors' attitude for example).

Our response:

We agree that the figure does not directly fit any of the hypotheses. We have therefore implemented the suggestion and plotted the change in theory-practice integration on the y-axis and the attitudes of the lecturers on the x-axis. We used a 2D density plot because scatter or point plots would be too cluttered. Further, we added density distributions of each of the two variables.

Minor aspects and typos

We corrected all minor aspects and typos according to your suggestions.
