# Supplementary material for: Using authentic representations of practice in teacher education: Do direct instructional and problem-based approaches really produce different effects?
Source: PLoS One. 2022 Sep 2;17(9):e0273988. doi: 10.1371/journal.pone.0273988 (PMC9439238; doi:10.1371/journal.pone.0273988)
Supplement: Attachment — Submitted filename: Review_PlosOne_December2021.docx [file pone.0273988.s002.docx]

The article “Using authentic representations of practice in teacher education: Do direct instructional

and problem-based approaches really produce different effects?” describes a large study in which student teachers analyze vignettes before and after receiving instruction on classroom management.

This study compares students who received the instruction in a problem-based approach to students who received the instruction in a direct instruction approach. Using Bayes factors the authors find that it seems not to depend on the instructional approach whether the students pay more selective attention, exhibit higher reflective thought or show more theory-practice integrations.

First of all, I congratulate the authors for a methodologically sound manuscript with an impressive study and sample size. However, I would like the manuscript to be further developed. In the following I describe main aspects that would improve the clarity of the manuscript.

Introduction

I think the summary of the reviews could be more concise, more to the point. There is no advantage of citing that researchers are "reinventing the wheel" (ll125f).

You claim to compare Problem Based Learning and Direct Instruction and your description of the methods you used fits these terms. However, in my particular understanding the definition of DI from Kirschner (2006) that you bring, would only define a “darstellende Stoffvermittlung”. In the current agreement of educational science, Direct Instruction names a specific instruction method that includes multiple phases.

The hypotheses are clear and could be tackled with this data. It would help the understanding of the results, if you already here make clear that you always test the sub-hypotheses in combination (and give a reason for it).

Method

You use the number of attended sessions, the number of articles read for these sessions and instructor’s attitude towards the instruction method as proxies/control variables. However, it remains unclear, what these proxies are really measuring. A more thorough discussion would be indicated.

From a pedagogical point of view I do not really find the task described in lines 329-331 as promoting learning. A good task (Arbeitsauftrag) should be structured more and be more elaborate.

I wondered whether each instructor had courses in both conditions. Please specify.

In section 3.4.1. there appeared again a short literature overview. I would shift this to the introduction above.

In section 3.4.2 I wondered why you tested for unidimensionality of the vignettes? Do you mean the raters score of reflective thought per vignette? Why did you only test for unidimensionality of reflective thought and not of selective attention as well?

When you report interrater reliability, please also specify how many cases have been rated by two (or even three) raters. Please also state what you did in cases in which the raters had different scores.

When reading the sentence in ll. 395 it was unclear to me what you meant by it. After reading part 3.5, I understood that you aimed at matching similar vignettes to pre- and posttest. Please clarify.

Results

Please make clearer, that you test the two sub-hypotheses of each dependent variable in combination. Currently, the indices of the hypotheses do not match and it is difficult to match the hypotheses and results. I would number the three main hypotheses with numbers from 1-3 and the different cases that you test (hypothesized direction, opposite direction, null hypothesis, unrestricted hypothesis) with letters from a-d for example.

Figure 2: Would it be possible to depict the raw data instead of the 12 data points here? It is not really interesting how the theory-practice integration differs by the vignettes, but more how it differs by DI vs PBL and how strongly students vary in that. Raw data per student would be very interesting.

Discussion

You state that you included the students’ willingness for effort and their attitude on readiness for reflection, but I did not see any results regarding this. Is this reported in the supplementary material.

You state “Students’ theory–practice integrations when analyzing classroom situations greatly increased from pretest to posttest.” Beforehand you ask the reader to be careful when comparing the scores between pre- and posttest, as the vignettes differ. I thus find that this conclusion is a bit far fetched. It could just be due to the different vignettes.

Supplementary Material

I very much liked that the supplementary material contains a knitted R markdown file containing all the code and results. I wondered why the authors put the data on the gesis server in a proprietary .sav format (maybe add a .txt or .csv file as well). Please also upload the data “delete.Rdata”, as this is vital to reproduce your code.

Language

I suggest a sound proofreading focusing of the clarity and precision of the language. Also please make use of the past tense, and use it consistently. You may also consider asking the writing experts at your institution. From my experience, this is always greatly improving a manuscript.

**Minor aspects:**

- Use key nouns consistently: for example, the manuscript uses “DI and PB methods”/“PB instruction”/“PB learning”/“PBL” → choose one. This also happens for “subject”/“student”/“teacher student” and “count”/“number”/“quantity” and “second semester”/“2^nd^ semester”/“teachers grade level (second)”. Please be very precise and consistent in the words you use.
- I would not number the overview of current research as “2.”, but put it as sub-sections of the Introduction.
- I found the following typos and obscurities:

Line 75/76: unclear how the reference belongs to the sentence, as this paragraph refers to Marsh & Mitchell (2014)

Line 117: unclear what “their” refers to

Line 118: “what’s more” → colloquial; at least write it out → “what is more”

Line 133: “the treatments” → which treatments are you referring to?

Line 143: “the authors” → imprecise. The authors of this study? (-> "we") vs. the authors of the former reviews (-> "the authors of the abovementioned reviews")

Line 150: "little robust evidence" 🡪 imprecise. I know what you mean, but I suggest you leave out 'robust' here.

Line 170: no full stop before the reference

Line 187-189: why does this sentence come at this place? It confused me, as the rest of the paragraph contains another description of prior research

Line 225: “toward instructors that” 🡪 “toward instructors who”

Line 223: “and predict” 🡪 “and can predict”

Line 273: “Participants were M_age = 21.01 (SD age = 2.36) years old on average” 🡪 “Participants were 21.01 (SD age = 2.36) years old on average”

Line 290: “8am” 🡪 “8 am”

In line 297 I suggest that you add a sentence that BFs range from 0 to infinity and that BFs << 1 point into the direction of the H0 and BFs >> 1 into the direction of H1.

Line 329: “i.e. vignettes” → “i.e., vignettes”

Line 409-410: sentence is not correct

Line 421: “we operationalize selective attention as situations” → it is an unclear operationalization. You do not use the situations, but you operationalize selective attention as “the ability of the participant to identify relevant topics”

Line 448: “the scale” → “a scale”

Line 465: As far as I understand, each vignette had a score from 0 to 3. Thus it is unclear to me how you calculate the mean score (or for what reason).

Line 489: reference incomplete

Line 506: “during the yearlong training, “ - leave out ‘yearlong’; at least you state in section 3.3 that the instructors started one semester before the study. inconsistent.

Line 510: “on the 6-point” → “on a 6-point”

Line 510 -512: please do not report only the range of points but also a mean. Maybe a Bayes Factor here would also help to judge whether there was really no difference. Was there a change from pre- to posttest?

Line 513: “we used it” → “we used instructor’s attitude”

Line 544: “.950-975” → “.950-.975”

Line 573-575: bad sentence structure

Line 613: the delta is not in percent, but in percentage points

Line 652-654: this belongs into the results section

In addition, you write in the ethics statement “(no appruval number), “ → “(no approval number).”

Furthermore, I found the use of past and present tense confusing, and suggest using this more consistently.

The text contains typos, careless errors and inconsistencies. I highly suggest, to let an English native speaker read over the text again. Please check more carefully for typos next time BEFORE submission!

I know that I expect a lot and I am fine if the authors do not consider each point for this work if they good reasons why this cannot be changed.

I hope my comments contribute to improve this work.

Best regards
